# Motor imagery-based brain-computer interface for differential diagnosis in prolonged disorders of consciousness

**DOI:** 10.3389/fnhum.2025.1695730

**Published:** 2025-11-27

**Authors:** Ping Liu, Qianqian Ge, Linghui Dong, Liqin Jiao, Shuai Han, Xiaoyang Kang, Haochong Wang, Jianghong He, Hao Zhang

**Affiliations:** 1 School of Rehabilitation, Capital Medical University, Beijing, China; 2 Department of Neurorehabilitation, China Rehabilitation Research Center, Beijing, China; 3 Department of Neurosurgery, Beijing Tiantan Hospital, Capital Medical University, Beijing, China; 4 Department of Neurocritical Care Rehabilitation, Closely Affiliated Medical Consortium Unit of China Rehabilitation Research Center, Beijing, China; 5 School of Life Sciences and Technology, Institute of Health and Rehabilitation Science, Xi’an Jiaotong University, Xi’an, Shaanxi, China

**Keywords:** prolonged disorders of consciousness, electroencephalography, motor imagery, brain-computer interface, assessment

## Abstract

**Introduction:**

Patients with prolonged disorders of consciousness (pDoC) present significant challenges to the assessment of consciousness. This study investigated the clinical utility of motor imagery-based brain-computer interface (MI-BCI) for discriminating consciousness levels in patients with pDoC.

**Methods:**

Thirty-one pDoC patients [12 with unresponsive wakefulness syndrome (UWS) and 19 in a minimally conscious state (MCS)] underwent EEG recordings during resting state and MI-BCI training. The analysis focused on relative power spectral density across five frequency bands (delta, theta, alpha, beta, gamma) in motor imagery-related regions (frontal and parietal cortices), along with BCI performance metrics (classification accuracy and attention indices).

**Results:**

We found that MCS patients exhibited multiband neural oscillation modulation during MI-BCI tasks, including slow-wave enhancement [(delta in frontal lobes (*p* = 0.003); theta in frontal (*p* = 0.026) and parietal lobes (*p* < 0.001)) and fast-wave suppression (alpha in frontal (*p* < 0.001) and parietal lobes (*p* = 0.049); beta in frontal (*p* = 0.014) and parietal lobes (*p* = 0.001); gamma in parietal lobes (*p* = 0.023)]. In contrast, UWS patients only showed localized parietal gamma enhancement (*p* = 0.042). Notably, the MCS group achieved significantly higher classification accuracy (55% vs. 38%, *p* = 0.02), and attention indices correlated moderately with CRS-R scores across all patients (Spearman’s ρ = 0.43, *p* = 0.02).

**Conclusion:**

The findings suggest that MI-BCI classification accuracy and attention indices may serve as auxiliary discriminators between UWS and MCS patients, with MCS patients demonstrating superior responsiveness to MI-BCI training.

## 1 Introduction

With advancements in medical science and emergency care capabilities, an increasing number of patients surviving severe strokes, traumatic brain injuries (TBI), or hypoxic-ischemic brain injuries now achieve vital signs stability, yet remain in prolonged states of impaired consciousness. When disorders of consciousness (DoC) persist beyond 28 days due to various etiologies, they are classified as prolonged disorders of consciousness (pDOC), encompassing both vegetative state (VS)/unresponsive wakefulness syndrome (UWS)–characterized by wakefulness without awareness and minimally conscious state (MCS), where intermittent behavioral evidence of conscious perception is detectable (Zhang and Ling, 2023; Giacino et al., 2018; Bruno et al., 2012). Patients in MCS exhibit a more favorable prognosis compared to those in UWS. Accurate assessment of consciousness levels is critical for determining prognosis and guiding multidisciplinary rehabilitation. Therefore, it is clinically imperative to reliably differentiate between UWS and MCS, as this distinction informs rehabilitation resource allocation, prognostic evaluation, and the development of personalized treatment protocols. However, patients with pDOC face significant diagnostic challenges due to severe functional impairments across multiple domains, including consciousness, language, cognition, and motor functions. These deficits render them incapable of reliable communication with the external environment. Clinically, it is often difficult to differentiate volitional behaviors from random, non-purposeful movements. Their behavioral responses frequently exhibit marked variability and inconsistency, which may compromise the accuracy of consciousness assessments using conventional behavioral scales. Furthermore, accurate assessment of therapeutic outcomes through clinical behavioral observations remains particularly challenging in these patients. Both international and national clinical guidelines and consensus statements on the diagnosis and management of pDoC recommend the standardized clinical application of the Coma Recovery Scale-Revised (CRS-R) to assess consciousness levels in patients with pDoC (Zhang and Ling, 2023; Giacino et al., 2018; Giacino et al., 2004). Recent advancements in neuroelectrophysiological and neuroimaging technologies have significantly deepened the understanding of pDoC. Electroencephalography (EEG) is an electrophysiological monitoring technique that records spontaneous electrical potential fluctuations in the brain. Both conscious states and psychological processes significantly influence EEG characteristics. Studies demonstrates that EEG can detect brain activation in patients who lack behavioral responses to verbal motor commands, establishing it as the gold standard for identifying covert consciousness (Owen, 2020; Pan et al., 2020). However, clinicians face significant challenges in precisely determining consciousness states due to the need for complex algorithmic analysis of EEG data.

With rapid technological advancements, brain-computer interface (BCI) technology, as a groundbreaking advancement in the field of neurorehabilitation, has emerged as a major research focus both domestically and internationally in recent years (Liu and Ushiba, 2022). Particularly, the motor imagery (MI)-based BCI paradigm has emerged as a prominent research due to its completely non-invasive characteristics and neuroplasticity-inducing properties (Pfurtscheller and Neuper, 2006; Khademi et al., 2023). MI involves the mental simulation of movement without physical execution, and when integrated with electroencephalography (EEG), it enables direct external device control by decoding neural signals into actionable commands—effectively bypassing damaged neuromuscular pathways (Mane et al., 2020). This technology demonstrates its core advantages through a dual-mechanism approach to functional recovery: primarily, the BCI system establishes an alternative communication channel for patients to interact with the external environment; secondarily, by decoding neural activity and providing real-time feedback, BCI training induces neuroplastic modifications that facilitate the repair and reorganization of impaired neural circuits (Mane et al., 2020). As a promising BCI paradigm, MI-BCI has demonstrated significant efficacy in neurorehabilitation applications, particularly in stroke recovery and Parkinson’s disease symptom management (Ji et al., 2021; Cuomo et al., 2022). Neuroimaging studies have elucidated the neurophysiological mechanisms underlying BCI-mediated therapeutic effects. Functional magnetic resonance imaging (fMRI) studies demonstrate that BCI-assisted rehabilitation enhances resting-state functional connectivity in the supplementary motor area and improves interhemispheric synchronization in stroke patients (Wang et al., 2019). Neurophysiological studies further reveal that MI-BCI can elicit activation patterns in the sensorimotor cortex, precuneus, and inferior parietal lobule that closely resemble actual movement execution (Nierhaus et al., 2021). During motor imagery tasks, acute stroke patients exhibited significant event-related desynchronization in alpha and beta frequency bands in the contralateral motor cortex corresponding to the imagined limb (Liu et al., 2024). These findings establish a robust theoretical framework for translating BCI technology into clinical practice.

In recent years, MI-BCI technology has emerged as an innovative tool for pDoC assessment, overcoming limitations of conventional behavioral methods. The seminal fMRI study by Owen et al. (2006) first demonstrated preserved MI activation patterns in a UWS patient, establishing MI as a sensitive marker for detecting covert awareness. These findings offer crucial insights for developing MI-BCI-based consciousness assessment protocols. However, while fMRI faces clinical limitations in pDoC patients due to severe neurological impairments and dynamic fluctuations in consciousness states, EEG-based MI-BCI offers millisecond-scale temporal resolution and superior bedside feasibility for serial consciousness monitoring.

Current applications of MI-BCI in pDoC face two critical challenges: (1) the representation of MI across consciousness levels (e.g., MCS vs. UWS) lacks systematic characterization; and (2) differential task responsiveness between MCS and UWS patients remains poorly understood, particularly in terms of BCI control capacity and its correlation with clinical assessments. To address these gaps, the current study employs EEG to systematically investigate neurophysiological differences during MI-BCI training in pDoC patients with varying consciousness states, aiming to optimize BCI-based consciousness state identification.

## 2 Materials and methods

### 2.1 Participants

Patients with pDOC were enrolled from the department of Neurocritical Care Rehabilitation at a closely affiliated medical consortium unit of China Rehabilitation Research Center from March to November 2024. The inclusion criteria were as follows: Diagnosis aligned with the Chinese Expert Consensus on Diagnosis and Treatment of Chronic Disorders of Consciousness; Age 18–65 years; Disease duration ≥ 3 months; Right-handedness; Written informed consent provided by legally authorized guardians. Exclusion criteria: Severe systemic comorbidities (including but not limited to cardiac, pulmonary, hepatic, or renal disorders) resulting in unstable vital signs; Severe visual and auditory pathway impairments, with clinical judgment of the simultaneous absence of visual and auditory startle responses; severe hand contractures or deformities precluding pneumatic glove application. Finally, a cohort of 31 pDOC patients were included in the study.

The CRS-R was administered to assess participants’ consciousness levels. All patients underwent five repeated assessments within a 2-week period, with the highest score recorded. Based on these scores, the 31 enrolled patients were stratified into two groups: UWS (*n* = 12) and MCS (*n* = 19). The UWS cohort comprised: TBI (*n* = 3), cerebral hemorrhage (*n* = 4), hypoxic-ischemic brain injury (*n* = 4), and large-area cerebral infarction (*n* = 1). Mean disease duration 6 ± 3.13 months; mean age 45.58 ± 11.49 years. The MCS cohort included: TBI (*n* = 9) and cerebral hemorrhage (*n* = 10). Mean disease duration 8.58 ± 5.15 months; mean age 48.2 ± 10.94 years. The two groups showed no statistically significant differences in age, sex distribution, or disease duration (Table 1). All enrolled participants received comprehensive rehabilitation therapy following the Chinese Expert Consensus on Rehabilitation of Prolonged Disorders of Consciousness (Zhang and Ling, 2023). The standardized protocol included: (1) physical therapy, (2) occupational therapy, (3) dysphagia management, and (4) traditional acupuncture therapy. This study was approved by Ethics Committee of the China Rehabilitation Research Center (Approval No. 2024-026-1). Since the participants were patients with disorders of consciousness lacking decision-making capacity, their legal guardians were fully informed of the study purpose and procedures. Written informed consent was obtained from all patients’ guardians prior to participation.

**TABLE 1:** Baseline demographic characteristics and the average number of successful completions in 15 MI trials for each group.

Variables	VS (*n* = 12)	MCS (*n* = 19)	*t*(χ^2^)	*p*
Age (years)	45.58 ± 11.49	48.2 ± 10.94	−0.619	0.542
Gender (male)	8/12	16/19	1.295	0.384
Disease duration (months)	6.08 ± 3.03	8.58 ± 5.15	−1.517	0.140
The average number of Successfully completed MI trails	5.75 ± 1.55	8.21 ± 2.15	−3.701	p<0.001

### 2.2 Experimental protocol

#### 2.2.1 The resting state (Rest)

Resting-state EEG data were collected from all subjects with a recording time of 5 min.

#### 2.2.2 The MI-BCI training

**FIGURE 1:**
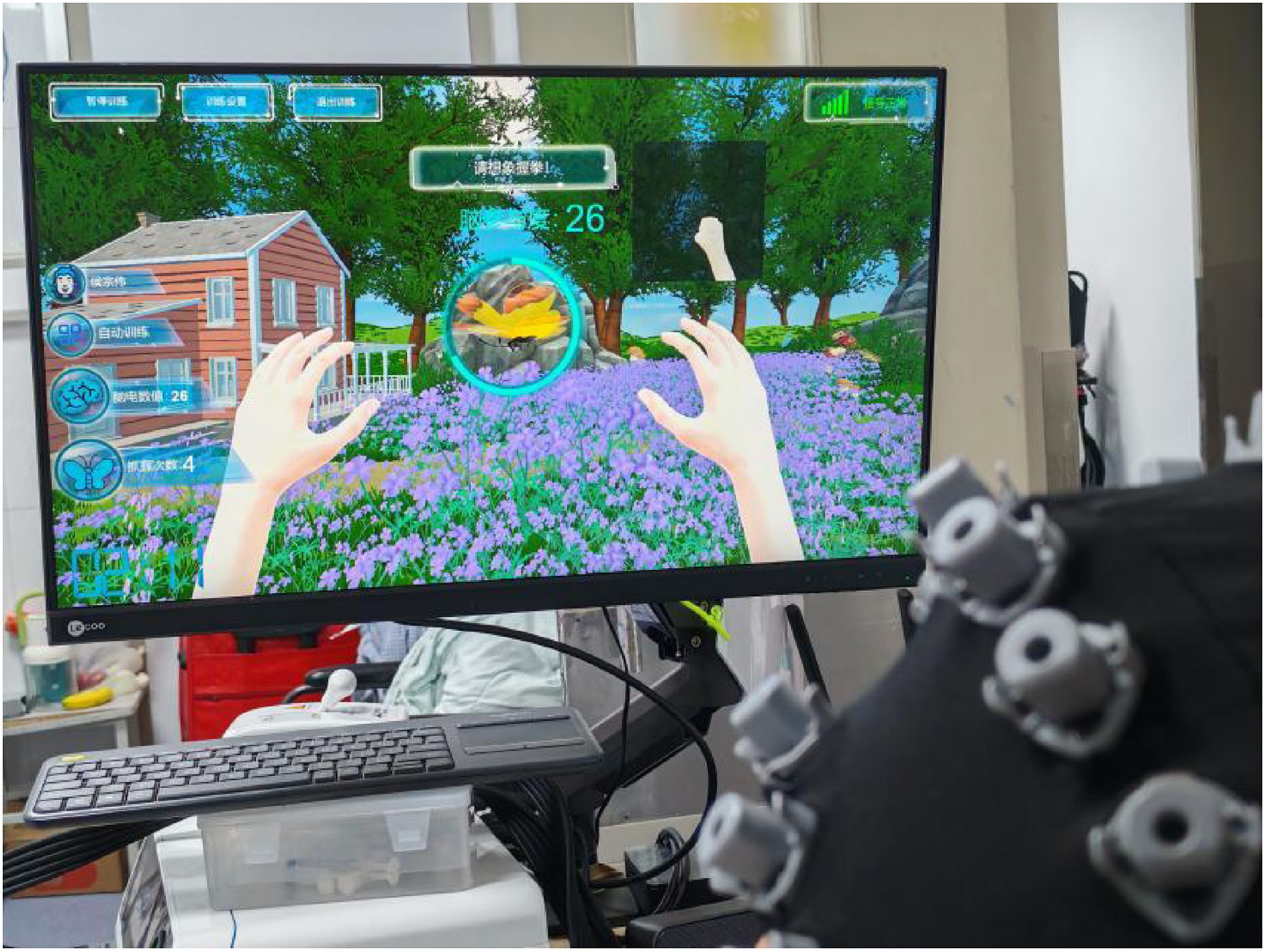
MI-BCI task interface. During the motor imagery task, the computer delivered an auditory command (“Please imagine fist-clenching”) accompanied by a corresponding text prompt (“Please imagine fist-clenching”) displayed in the upper-central area of the screen, along with an animated short film demonstrating the imagined action.

MI-BCI was implemented using the hand rehabilitation training system (ZhenTec–H1, ZhenTec Intelligence Technology Co., Ltd., Xi’an, China). Every participant positioned the wheelchair opposite a computer screen was fitted with an EEG cap and a pneumatic glove was weared on the right hand. During the training, participants focused on a screen with a resolution of 1,920 × 1,080 pixels. An auditory command (“Please imagine clenching your fist”) was then played at 85 decibels, which was the system’s maximum volume. This was accompanied by a corresponding text prompt displayed in the upper-central area of the screen, along with an animated short film demonstrating the action to be imagined, as shown in Figure 1. Participants were instructed to mentally simulate the action of the right-hand fist-clenching in response to the cue, while their neurological engagement levels (measured as an attention index) were monitored in real time. When the level of attention index exceeds the preset threshold, the soft rehabilitation glove system provides feedback by assisting the patient in completing a grasping motion, accompanied by rewarding audiovisual feedback (e.g., “Great job!”). If the threshold is not met, the system withholds assistance but delivers an encouraging verbal prompt (e.g., “Keep on fighting!”). Each participant underwent 15 training trials. (Prior to formal BCI training, all patients received detailed explanations and demonstrations of the protocol, followed by 5 practice trials to mitigate potential practice effect bias. Data from these practice trials were excluded from formal analysis).

### 2.3 Data recording and preprocessing

#### 2.3.1 Data recording

EEG data were recorded synchronously during Rest and MI-BCI training using a 32-channel wireless EEG recorder amplifier (ZhenTec-NT1, ZhenTec Intelligence Technology Co., Ltd., Xi’an, China). The EEG scalp electrodes were arranged according to the international 10-10 system. The reference electrode is positioned at CPz, and the ground electrode is positioned at AFz. All electrodes were Ag/AgCl semi-dry, made from a highly absorbent sponge wetted with 3%-NaCl solution. Additional electrooculography (EOG) collect relevant voltages using Ag/AgCl electrodes and conductive hydrogel. Resting-state EEG signals were sampled at 500 Hz, while MI-BCI training data were acquired at 250 Hz. This lower sampling rate for the MI-BCI data was chosen for two reasons. First, as the neurophysiological correlates of MI, such as sensorimotor rhythms (e.g., mu and beta rhythms) are typically below 30 Hz, and given the relatively long duration of MI neural responses, a 250 Hz sampling rate—sufficient to capture frequencies up to 125 Hz in accordance with the Nyquist theorem—adequately covers the frequency range of interest while minimizing aliasing risk. Second, this rate effectively reduces computational load and data storage requirements without compromising signal integrity, which aligns with common practice in contemporary MI-BCI studies (Zhu et al., 2025; Mathiyazhagan and Devasena, 2025). Throughout the experiment, all electrode impedances were maintained below 20 kΩ.

#### 2.3.2 Data preprocessing

EEG data were preprocessed using Matlab2021b and EEGlab toolbox. First, the EEG recordings were bandpass filtered 1–45 Hz using a 6th-order Butterworth filter, and 50 Hz notch filter was applied using a ERPLAB filter, then each channel of intercepted EEG was decomposed into five sub-bands of interest: delta (1–4 Hz), Theta (4–8 Hz), Alpha (8–15 Hz), Beta (15–30 Hz), and Gamma (30–45 Hz) via the band-passed filter. All 30 channels were grouped into 4 brain regions: frontal (Fp1/2, F3/4, Fz, FC3/4, FCz, F7/8, FT7/8), parietal (C3/4, Cz, CP3/4, CPz, P3/4, Pz), temporal (T3/4, TP7/8, T5/6), and occipital (O1/2, Oz). The data were then visually inspected to identify and interpolate bad channels, and extreme values were removed. Independent component analysis (ICA) was subsequently applied for further artifact attenuation. To ensure the quality and comparability of the EEG data, a rigorous signal quality assessment was performed. The Signal-to-Noise Ratio (SNR) was calculated for each subject before and after the automated artifact removal pipeline. The SNR was defined as the ratio of the mean power in the 8–30 Hz band (encompassing mu and beta rhythms) during task periods to that during pre-stimulus baseline periods. The average SNR across all participants was −2.1 ± 0.8 dB prior to preprocessing. After the full preprocessing pipeline (comprising filtering, bad epoch rejection, and independent component analysis), a significant increase in the mean SNR to 5.4 ± 1.2 dB was observed (paired *t*-test, *t* = 25.6, *p* < 0.001). Finally, the EEG data were common average re-referenced, non-EEG electrodes (e.g., EOG) were excluded, and the continuous data were segmented into a window of 5 s, with segments containing amplitudes exceeding ± 75 μV in any channel being discarded.

### 2.4 Analysis methods

#### 2.4.1 Power spectral density

PSD is an effective method to differentiate between noise and features in a signal by making aspectral representation of the power distribution of its frequency components. We use Welch’s method (Astfalck et al., 2024) to compute PSD. Additionally, to enhance spectral estimation performance, we applied a sliding Hamming window with a length of 500 ms and an overlap of 250 ms. Firstly, we calculated the FFT of each windowed data segment and obtained its periodogram. Secondly, the average value of the periodic graph of all windowed data segments was used as the final spectral estimate of the signal. Then the PSD results of each frequency band were normalized to obtain the relative PSD of one band to the whole frequency band.


$$P\,o\,w\,e\,r_{r\,e\,l\,a\,t\,i\,v\,e}=\frac{\sum_{f=f_{1}}^{f=f_{2}}P\,o\,w\,e\,r\,\left(f\right)}{\sum_{f=f_{L}}^{f=f_{H}}P\,o\,w\,e\,r\,\left(f\right)}$$

where [f_*L*_, f_*H*_] = [1, 45] and [f_1_, f_2_] is determined by the frequency sub-band selected.

#### 2.4.2 BCI classification accuracy

The MI-BCI system’s motion intention classification accuracy was quantified by the success rate of grasping tasks, defined as the ratio of successfully completed control trials to the total number of attempted control trials. This study compared the classification accuracy between the two groups during MI tasks.

#### 2.4.3 Attention index

The MI-BCI system computes an attention index derived from the energy-weighted ratio of beta-to-alpha band power (*R* = Eβ/Eα, normalized to a 0–100 scale) in EEG signals recorded at the prefrontal (Fp1) electrode during motor imagery (MI) tasks (Yuan et al., 2021). In this study, the attention index threshold was set to 50. When the moving average of the attention index exceeds this threshold, the system triggers the soft rehabilitation glove to assist the patient in performing a grasping movement, thereby establishing closed-loop feedback. Additionally, this study examined the correlation between participants’ attention indices and their corresponding CRS-R scores.

### 2.5 Statistical analysis

Statistical analysis was performed using SPSS 26.0 software. The data of relative power were expressed as quartiles. The EEG data related to motor imagery in the frontal and parietal lobes/5 EEG frequency bands (Delta, Theta, Alpha, Beta, and Gamma) during the resting state and MI-BCI were analyzed independently for the two patient groups (UWS group and MCS group). The Wilcoxon rank-sum test was used for between-group comparisons, while the Wilcoxon signed-rank test was used for within-group comparisons. The data of classification accuracy rates were presented as mean ± standard deviation. The classification accuracy rates of MI-BCI between the two groups were compared using independent samples *t*-tests. Spearman’s rank correlation coefficient was employed to analyze the association between attention indices during MI-BCI tasks and CRS-R scores. The statistical significance threshold was set at *p* < 0.05.

## 3 Results

### 3.1 Differences in EEG features and intervention effects between Rest and the MI-BCI task state

The analysis of the relative power changes in the five EEG frequency bands (Delta, Theta, Alpha, Beta, and Gamma) in the frontal and parietal lobes during Rest and MI-BCI hand function training for the two groups of participants showed that (Table 2).

**TABLE 2:** Differences in EEG features and intervention effects in the frontal and parietal lobes between UWS and MCS patients under Rest and MI-BCI task state.

Brain region/ frequency band	Group	Rest	MI-BCI	*Z*	*p*	Delta (Δ)
Frontal delta	MCS	0.35 (0.27, 0.44)	0.43 (0.37, 0.51)	24	0.003	0.09 (0.00, 0.13)
	VS	0.44 (0.38, 0.51)	0.45 (0.38, 0.53)	37	0.91	−0.01 (−0.06, 0.09)
	Z	−2.06	−0.14			−1.60
	p	0.039	0.889			0.109
Parietal delta	MCS	0.36 (0.23, 0.42)	0.38 (0.32, 0.44)	61	0.182	0.02 (−0.03, 0.13)
	VS	0.43 (0.37, 0.55)	0.42 (0.32, 0.47)	56	0.204	−0.05 (−0.07, 0.04)
	Z	−2.32	−0.82			−1.16
	p	0.02	0.412			0.248
Frontal theta	MCS	0.28 (0.23, 0.32)	0.36 (0.29, 0.39)	40	0.026	0.06 (0.00, 0.15)
	VS	0.25 (0.24, 0.29)	0.29 (0.26, 0.35)	24	0.266	0.05 (−0.02, 0.11)
	Z	−0.46	−0.94			0.22
	p	0.646	0.346			0.823
Parietal theta	MCS	0.24 (0.21, 0.28)	0.36 (0.31, 0.39)	0	< 0.001	0.13 (0.05, 0.16)
	VS	0.25 (0.23, 0.27)	0.30 (0.27, 0.35)	18	0.11	0.07 (0.02, 0.10)
	Z	−0.66	−1.27			−1.16
	*p*	0.509	0.205			0.248
Frontal alpha	MCS	0.22 (0.17, 0.26)	0.12 (0.11, 0.17)	176	< 0.001	−0.08 (−0.13, −0.03)
	VS	0.18 (0.12, 0.24)	0.15 (0.07, 0.20)	48	0.519	−0.03 (−0.13, 0.07)
	Z	−1.47	−0.18			−1.24
	*p*	0.141	0.857			0.216
Parietal alpha	MCS	0.24 (0.17, 0.26)	0.16 (0.15, 0.19)	144	0.049	−0.03 (−0.09, 0.00)
	VS	0.20 (0.11, 0.23)	0.17 (0.12, 0.22)	42	0.85	0.03 (−0.12, 0.08)
	Z	−1.47	−0.34			−0.95
	p	0.141	0.734			0.341
Frontal beta	MCS	0.10 (0.08, 0.15)	0.04 (0.04, 0.05)	155	0.014	−0.07 (−0.08, −0.03)
	VS	0.09 (0.07, 0.12)	0.07 (0.03, 0.20)	32	0.622	0.02 (−0.05, 0.07)
	Z	−1.1	−1.1			−1.97
	p	0.269	0.269			0.049
Parietal beta	MCS	0.15 (0.12, 0.18)	0.05 (0.03, 0.06)	171	0.001	−0.10 (−0.14, −0.06)
	VS	0.12 (0.05, 0.14)	0.11 (0.03, 0.17)	36	0.85	0.02 (-0.06, 0.06)
	Z	−2.15	−1.1			−2.45
	*p*	0.032	0.269			0.014
Frontal gamma	MCS	0.01 (0.01, 0.02)	0.01 (0.00, 0.01)	140	0.073	−0.00 (−0.02, 0.00)
	VS	0.01 (0.00, 0.03)	0.03 (0.01, 0.05)	16	0.077	0.01 (−0.00, 0.04)
	Z	−0.94	−1.98			−2.33
	*p*	0.346	0.048			0.020
Parietal gamma	MCS	0.01 (0.01, 0.03)	0.00 (0.00, 0.01)	151	0.023	−0.01 (−0.02, −0.00)
	VS	0.01 (0.01, 0.01)	0.03 (0.01, 0.06)	13	0.042	0.01 (−0.00, 0.05)
	Z	−0.9	−2.54			−2.90
	*p*	0.367	0.011			0.004

#### 3.1.1 Between-group differences

Under the Rest condition, the delta band energy in the frontal and parietal lobes of the MCS group was significantly lower than that of the UWS group (frontal lobe *p* = 0.039, parietal lobe *p* = 0.02); the beta band energy in the parietal lobe of the MCS group was significantly higher than that of the UWS group (*p* = 0.032). Under the MI-BCI task state, the gamma band energy in the frontal and parietal lobes of the MCS group was significantly lower than that of the UWS group (frontal lobe *p* = 0.048, parietal lobe *p* = 0.011).

#### 3.1.2 Within-group intervention effects

**FIGURE 2:**
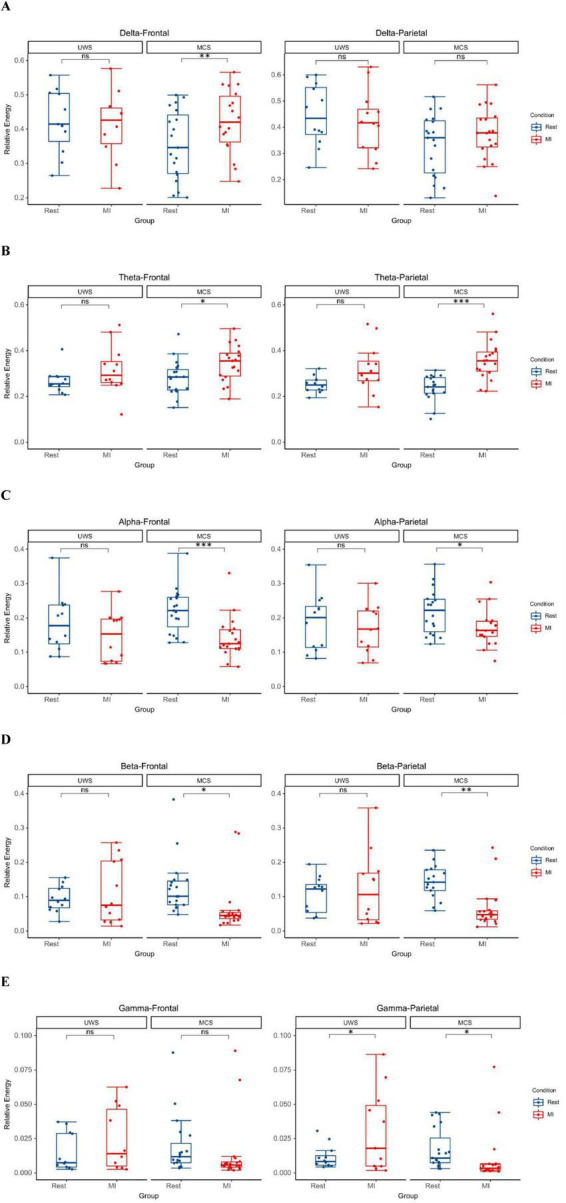
The MI-BCI intervention induced a trend of increased low-frequency (delta/theta) power accompanied by decreased high-frequency (alpha/beta/gamma) power in the MCS group, whereas the UWS group primarily exhibited enhanced gamma band oscillations in parietal lobes. **(A)** In MCS patients, MI-BCI resulted in a significant increase in frontal lobes delta power (*p* = 0.003). **(B)** In MCS patients, MI-BCI resulted in a significant increase in theta power in the frontal and parietal lobes (frontal: *p* = 0.026, parietal: *p* < 0.001). **(C)** In MCS patients, MI-BCI resulted in a significant decrease in alpha power in the frontal and parietal lobes (frontal: *p* < 0.001, parietal: *p* = 0.049). **(D)** In MCS patients, MI-BCI resulted in a significant decrease in beta power in the frontal and parietal lobes (frontal: *p* = 0.014, parietal: *p* = 0.001). **(E)** In MCS patients, MI-BCI resulted in a significant decrease in parietal lobes gamma power (*p* = 0.023), whereas in UWS patients, it caused a significant increase in parietal lobes gamma power (*p* = 0.042). *p < 0.05; **p < 0.01; ***p < 0.001.

For the MCS group, MI-BCI intervention led to a significant increase in delta (*p* = 0.003) and theta (*p* = 0.026) power in the frontal lobe, while alpha (*p* < 0.001) and beta (*p* = 0.014) power decreased significantly. In the parietal lobe, theta power increased significantly (*p* < 0.001), while alpha (*p* = 0.049), beta (*p* = 0.001) and gamma (*p* = 0.023) power decreased significantly. For the UWS group, MI-BCI intervention only resulted in a significant increase in gamma band power in the parietal lobe (*p* = 0.042) (Figures 2A–E).

#### 3.1.3 Between-group differences in intervention effects (comparison of the changes between MI-BCI and Rest)

Significant differences in the changes of beta (frontal lobe p = 0.049, parietal lobe *p* = 0.014) and gamma (frontal lobe *p* = 0.020, parietal lobe *p* = 0.004) band power in the frontal and parietal lobes were observed between the two groups, while no significant differences were found in the other frequency bands.

#### 3.1.4 EEG topographic maps of PSD

**FIGURE 3:**
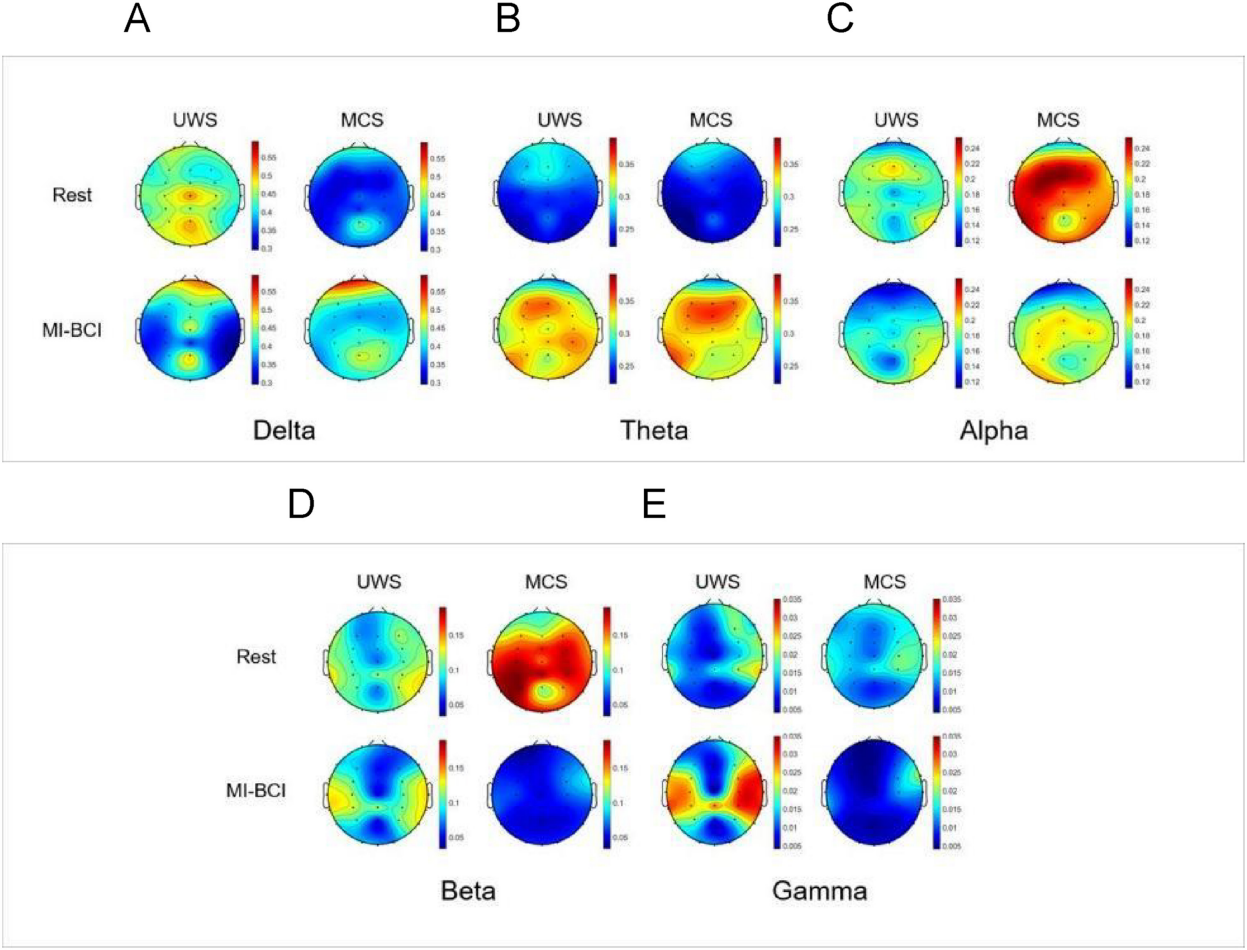
EEG topographic maps of spectral power distribution in pDoC patients during Rest vs. the MI-BCI Intervention. **(A)** Delta band (1–4 Hz): during Rest, MCS group showed significantly lower delta power in the frontal-parietal regions compared to UWS group (*p* < 0.05), while during the BCI intervention, the MCS group showed increased delta power specifically in the frontal regions (*p* < 0.01), resulting in attenuated intergroup differences following the intervention. **(B)** Theta band (4–8 Hz): during the BCI intervention, there was a significant increase in theta band energy in the whole brain of patients with MCS (*p* < 0.05).**(C)** Alpha band (8–13 Hz): during the BCI intervention, there was a significant reduction in alpha band energy in the frontoparietal regions (*p*< 0.01)in MCS group.**(D)** Beta band (13–30 Hz): during Rest, MCS group showed significantly higher beta power in the parietal regions compared to UWS group (*p* < 0.05), while during the BCI intervention, there was a significant reduction in beta band energy in the frontoparietal regions (*p*< 0.05) in MCS group. **(E)** Gamma band (30–45 Hz): during the BCI intervention, the gamma band energy demonstrated a significant increase in the parietal regions in UWS group and a significant reduction in the parietal regions in MCS group (*p*< 0.05).

For comparative analysis of Rest versus MI-BCI task, we visualized relative power distributions across 30 whole-brain channels to characterize frequency band-specific spatial distribution in UWS and MCS groups (Figures 3A–E).

### 3.2 Different features of MI-BCI are associated with levels of consciousness

#### 3.2.1 The classification accuracy

**FIGURE 4:**
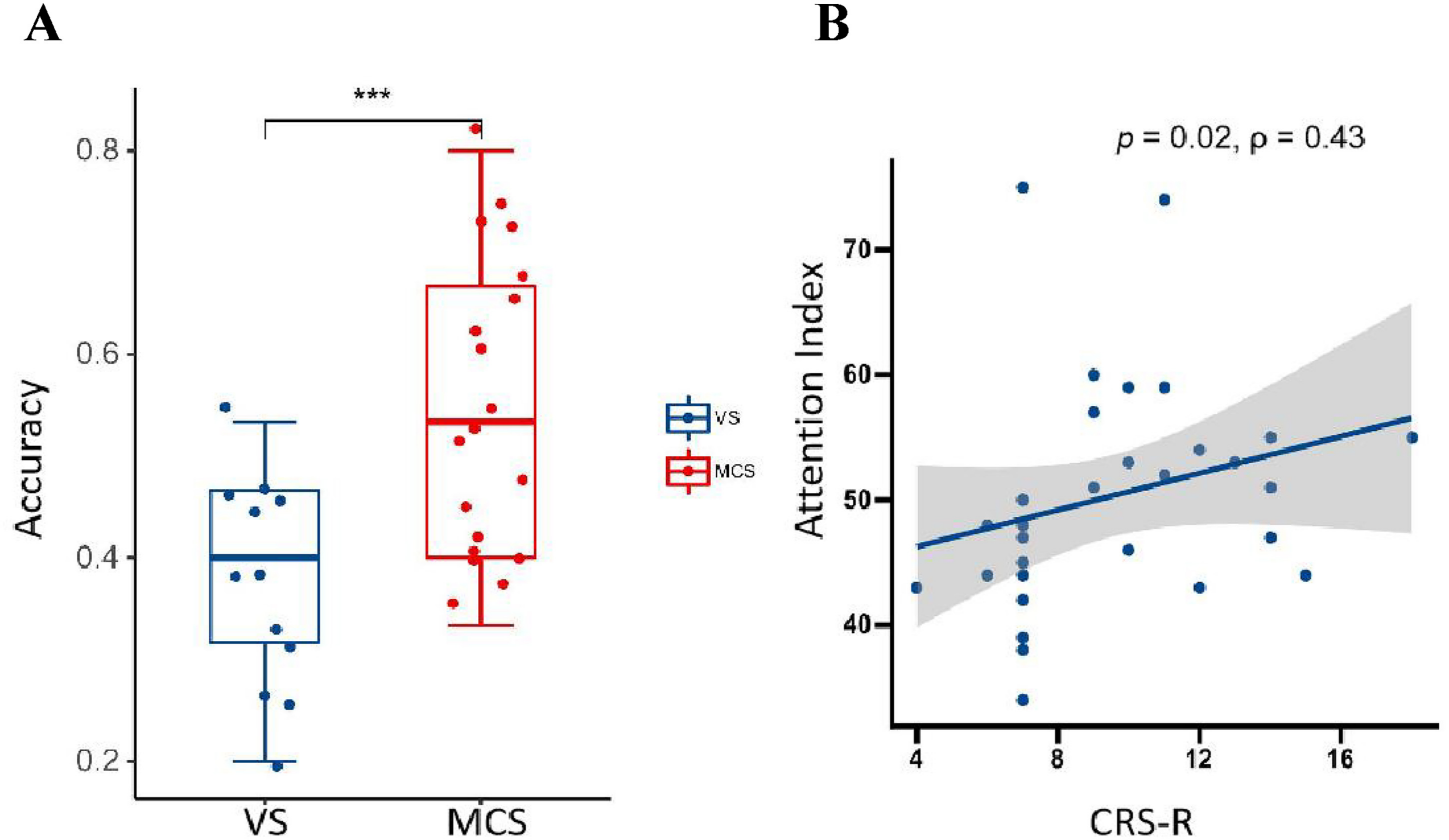
**(A)** MI-BCI classification accuracy between the two groups during MI tasks (*p*< 0.001). **(B)** Correlation between attention index and CRS-R scores. It could be seen that a moderately strong positive correlation exists between attention indices and CRS-R scores. The shaded area represents the 95% confidence interval (ρ = 0.43, *p* = 0.02). ***p < 0.001.

A comparison of MI-BCI classification accuracy between MCS and UWS groups revealed that the accuracy rate was 55 in the MCS group and 38% in the UWS group. The MCS group demonstrated significantly higher classification accuracy than the UWS group, with the difference reaching statistical significance (*p* = 0.00092) (Figure 4A).

#### 3.2.2 The relationship between participants’ attention indices and their corresponding CRS-R scores

The correlation analysis between attention index during motor imagery and CRS-R scores revealed that a moderate positive correlation was observed between the attention indices and CRS-R scores (ρ = 0.43, *p* = 0.02) (Figure 4B).

## 4 Discussion

This study investigated EEG responses during MI-BCI training in 31 pDoC patients, revealing consciousness-level-dependent neural engagement patterns. The key findings demonstrated distinct spectral response profiles between UWS and MCS patients, with MCS patients exhibiting enhanced neuromodulatory capacity and superior classification accuracy.

### 4.1 Consciousness-level-dependent neural engagement during MI-BCI tasks

MCS patients exhibited significantly lower resting-state delta power in frontoparietal regions (*p* < 0.05) and higher parietal beta power in the parietal lobe (*p* = 0.032) compared to UWS. This finding are consistent with previous studies showing that UWS patients typically display enhanced delta activity, with increased delta oscillations positively correlating with the degree of consciousness impairment (Duszyk-Bogorodzka et al., 2022). Moreover, studies have demonstrated that the beta/delta power ratio shows a significant negative correlation with the severity of consciousness impairment (Lutkenhoff et al., 2022). During MI-BCI, MCS patients displayed multi-band coordination (alpha/beta suppression with theta/delta enhancement) across frontal-parietal regions, while UWS patients exhibited only localized gamma bursts in parietal lobes. These findings demonstrate that MCS patients retain functional neuromodulatory capabilities, whereas UWS patients maintain only residual sensory processing with impaired higher-order integration. Consistent with existing evidence, gamma oscillations are associated with sensory signal processing in localized sensorimotor cortices (Szurhaj et al., 2005; Muthukumaraswamy, 2010). The observed intergroup differences suggest that MCS patients generally exhibiting higher levels of EEG activity and improved sensorimotor rhythm regulation compared to UWS patients.

Quantitative EEG analysis revealed increased theta power and decreased alpha/beta power in the frontoparietal network of MCS patients, potentially reflecting attentional and cognitive processing. Previous research has demonstrated that theta oscillations and the theta/beta power ratio may indicate modulations in attentional and cognitive states during interventions, particularly involving frontal executive function circuits and parietal associative cortex integration (Pomper and Ansorge, 2021; Kahya et al., 2022). Consistent with alpha’s inhibitory role (Klimesch, 2012), reduced alpha power marks the rest-to-task transition, with frontal alpha suppression showing an inverse correlation with attention allocation (Bagherzadeh et al., 2020). Extensive evidence has also established that motor execution characteristically suppresses alpha and beta oscillations in the sensorimotor cortex (Wilson et al., 2014). Comparatively, the observed frontal delta power increase (*p* = 0.003) may reflect task-induced fatigue. These findings indicate MI-BCI-induced functional reorganization of frontoparietal networks in MCS patients, with frontal regions potentially modulating task execution and parietal areas contributing to sensorimotor integration. The observed task-related sensorimotor activation and sustained attention in MCS patients suggest preserved higher-order cognitive processing capabilities. This finding aligns with Bareham et al. (2020), who demonstrated that functional network reorganization in pDoC patients precedes behavioral recovery, with theta/alpha modulation serving as a predictor of CRS-R improvement. Specifically, cortical activation may initially appear as global theta synchronization, followed by localized alpha modulation as brain function recovers. Moreover, quantitative EEG studies have shown that features such as fronto-parietal connectivity and inter-hemispheric communication—similar to the spectral features modulated in our MCS group—are robust predictors of both consciousness level and recovery potential (Toppi et al., 2024).

### 4.2 Differences in BCI-specific metrics between the two patient groups

This study further revealed that the MCS group demonstrated significantly higher MI-BCI classification accuracy than the UWS group (55% vs. 38%, *p* = 0.00092), indicating that patients with higher consciousness levels more readily achieve closed-loop feedback through MI. The consistency between attention indices during MI tasks and clinical CRS-R scores (positive correlation: ρ = 0.43, *p* = 0.02) implies that MI-BCI may enhance attention network connectivity and serve as an auxiliary consciousness assessment tool.

Furthermore, our findings reinforce the emerging consensus that MI-BCI—by directly accessing neural correlates of volitional MI and bypassing the need for motor output, can serve as a sensitive tool for detecting covert consciousness in patients who lack overt behavioral responses. The case of Patient 28 in our cohort—who achieved a classification accuracy of 73% despite absence of command-following behavior—exemplifies this potential. Such patients may in fact possess preserved cognitive motor integration and attentional resources that are undetectable through bedside observation and standard behavioral assessments alone. This aligns with existing literature on BCI paradigms (Galiotta et al., 2022; Edlow and Menon, 2024). Thus, MI-BCI not only complements existing diagnostic tools but may also reduce misdiagnosis rates by providing a neural communication channel for patients who are conscious yet behaviorally non-responsive.

### 4.3 Comparison with other BCI paradigms

Different BCI paradigms have been developed to assess residual cognitive and motor functions in patients with DoC, each requiring varying levels of cognitive engagement. Owen et al. demonstrated that a patient in the vegetative state could perform MI tasks (“playing tennis” or “walking through a house”) in fMRI, providing early evidence for covert command following (Owen et al., 2006). Monti et al. have even reported a case of a vs. patient who was able to answer several yes-or-no questions using two different mental imagery tasks (he was instructed to respond “yes” using one type of imagery task, such as motor imagery or spatial imagery, and “no” using the other) (Monti et al., 2010). Coyle et al. used two MI tasks—imagining right-hand gripping and toe movement—in four MCS patients, showing significant cortical activation during feedback training (Coyle et al., 2015). Furthermore, Schiff et al. confirmed the central role of BCI in identifying “covert consciousness,” asserting that MI-BCI can serve as a sensitive tool for detecting covert consciousness and cognitive motor dissociation (CMD) in patients who lack overt behavioral responses (Schiff et al., 2024). The ability of MI-BCI to identify covert consciousness has gained increasing recognition as a transformative approach in DoC assessment (Young et al., 2024).

In addition to MI–based BCIs, other paradigms such as the P300 have been used to detect covert awareness in patients with DoC. Lulé et al. employed an auditory P300 paradigm presenting simple verbal stimuli (“yes,” “no,” “stop,” “go”) and found that one MCS and one locked-in patient produced reliable command-following responses (Lulé et al., 2013). To enhance stimulus salience, Wang et al. designed an audio-visual hybrid BCI allowing DoC patients to select numbers according to combined auditory and visual cues, achieving higher accuracy than unimodal paradigms (Wang et al., 2015). In addition to visual or auditory paradigms, Spataro et al. (2022) used the vibrotactile P300 BCI to assess the command following. These findings highlight that P300-based BCIs probe attention and information-processing mechanisms and can complement MI-BCIs in identifying residual cognition when volitional MI is limited.

These studies indicate that MI-BCI and P300-BCI offer complementary strengths for detecting residual consciousness. MI-BCI, reliant on higher-order volition, is more feasible for MCS patients, whereas P300-BCI more effectively identifies residual cognition in UWS patients. Consequently, future integration of passive and active BCI paradigms could further enhance the detection of covert consciousness.

### 4.4 Challenges in pDoC and future directions for MI-BCI research

Existing evidence demonstrates disrupted effective connectivity between cortical regions in pDoC (Rosanova et al., 2012). The mesocircuit hypothesis posits that the core pathological mechanism in DoC involves widespread deafferentation, consistently manifesting as reduced sensorimotor cortex activity (Di Perri et al., 2016; Coulborn et al., 2021). Furthermore, previous studies have established that impaired MI performance in chronic spinal cord injury patients is associated with movement attempt-induced sensorimotor deactivation and afferent interruption (Owen et al., 2006; Müller-Putz et al., 2007). This mechanism may similarly explain the degraded MI performance observed in pDoC patients, likely resulting from either sensorimotor cortex hypoactivation or impaired sensory input due to thalamocortical pathway damage. Consequently, despite these promising findings, the achieved classification accuracy remained below the threshold required for clinical application (Nagarajan et al., 2024). This discrepancy may reflect a temporal misalignment between the system’s fixed 5-s analysis window and delayed neurophysiological responses in pDoC patients. Preliminary observations under current experimental conditions suggest that some patients exhibit delayed response patterns, with attention indices peaking approximately 3–5 s after MI cue offset. Implementing adaptive windowing (e.g., 8–10 s) or delayed-response algorithms may improve performance by accommodating these DoC-specific processing delays.

While BCI applications in DoC remain experimental (Annen et al., 2020), we observed significant interindividual variability in BCI performance. The current study’s limitations include a small sample size that may constrain statistical power and a lack of long-term follow-up data, both of which warrant further investigation. Future research directions should: (1) optimize BCI parameters (e.g., implementing adaptive time windows and deep learning algorithms); (2) expand sample sizes; and (3) conduct longitudinal intervention studies in patient subgroups achieving ≥ 55% BCI accuracy while monitoring long-term functional outcomes.

## 5 Conclusion

This study reveals frequency-specific neural response patterns in pDoC patients during MI-BCI training. EEG analysis confirmed training-induced cerebral activation, while MI-BCI metrics (classification accuracy and attention indices) may facilitate differentiation between MCS and UWS, offering a more portable and clinically feasible consciousness assessment approach. Future research could incorporate combined active-passive training for MCS patients and intensive multisensory stimulation for UWS patients to further track dynamic changes in brain rhythms linked to consciousness.

## Data Availability

The original contributions presented in this study are included in this article/supplementary material, further inquiries can be directed to the corresponding authors.
